# Training to Support ePortfolio Users During Clinical Placements: a Scoping Review

**DOI:** 10.1007/s40670-022-01583-0

**Published:** 2022-06-30

**Authors:** Sofie Van Ostaeyen, Mieke Embo, Tammy Schellens, Martin Valcke

**Affiliations:** 1 Department of Educational Studies, Faculty of Psychology and Educational Sciences, Ghent University, Henri Dunantlaan 2, 9000 Ghent, Belgium; 2 Artevelde University of Applied Sciences, Expertise Network Health and Care, Voetweg 66, 9000 Ghent, Belgium

**Keywords:** ePortfolio, Clinical placements, Training, Higher education, Healthcare education, Scoping review

## Abstract

ePortfolios are frequently used to support students’ competency development, and teachers’ and clinical mentors’ supervision during clinical placements. User training is considered a critical success factor for the implementation of these ePortfolios. However, there is ambiguity about the design and outcomes of ePortfolio user training. A scoping review was conducted to consolidate evidence from studies describing the design of ePortfolio user training initiatives and their outcomes. The search yielded 1180 articles of which 16 were included in this review. Based on the results, an individual, ongoing training approach which grounds in a fitting theoretical framework is recommended.

**Supplementary Information:**

The online version contains supplementary material available at 10.1007/s40670-022-01583-0.

## Introduction

Over the past decades, healthcare education has evolved from the traditional knowledge-based approach to a competency-based approach in an attempt to improve the quality of education, patient outcomes, and societal accountability [1, 2]. Competency-based education is outcome based. It fundamentally focuses on achieving graduate competence and is organized around a framework of competencies [3, 4]. Observable behaviour is the primary source to evidence mastery and assessment is related to students’ ability to demonstrate the achievement of competency standards [5]. Work-based learning or learning in clinical placements is an essential feature of this competency-based approach [6]. During clinical placements, students experience real-life care provision scenarios, allowing them to put theory into practice [7, 8]. This offers the opportunity to develop competencies in an authentic clinical environment [6, 9].

Digital or ePortfolios are tools often used to support competency development and assessment during clinical placements [10–13]. They give students the opportunity to document their learning process and demonstrate clinical competence [14, 15]. This information is referred to as ‘artefacts’ and can be stored in many media formats (e.g. text, images, video or audio) [16, 17]. The building of this artefact collection during practice facilitates reflection on performance and competency development, and reduces the gap between theory (classroom) and practice (clinical placement) [15]. In addition, ePortfolios support teachers (employed by an educational institution) and clinical mentors (employed in a workplace setting) in their supervisory role by providing them a tool to give feedback on learning activities and assess students’ performance and competency development during clinical placements [18, 19].

To ensure optimal use of ePortfolios, they must be implemented in a well-considered way and barriers that reduce the positive effects of ePortfolio use or hinder students, teachers or clinical mentors’ motivation to use the tool should be addressed [20, 21]. ePortfolio user training responds to these barriers and thus is considered as critical for successful ePortfolio implementation [22]. Educational programs that adopt ePortfolios recognize the need for user training and include it as part of the implementation phase [23, 24]. However, a review conducted by Tochel et al. [25] pointed out that training and support initiatives are rarely evaluated in ePortfolio studies. Related research is fragmented and an integrated overview of ePortfolio user training with its outcomes is missing. This causes ambiguity about the design of such training initiatives. This study aims to fill this gap by consolidating evidence from studies describing ePortfolio user training initiatives and their outcomes.

In order to achieve this aim, a scoping review was considered the most appropriate research method. A scoping review shares characteristics with a systematic review since both implement the following quality criteria: systematic, transparent and replicable [26]. Where a systematic review tries to respond to specific questions, a scoping review rather helps to identify, map, report and discuss a broader perspective on a phenomenon (e.g. ePortfolio user training) [27–29].

## Methods

The five-stage framework proposed by Arksey and O’Malley [30] was used to guide the review: (1) identifying the research question, (2) identifying relevant studies, (3) selecting studies, (4) charting the data, and (5) collating, summarizing and reporting the results.

### Identifying the Research Question

The following research question was stated in order to encompass the fragmented research on ePortfolio user training: What is known about the design and outcomes of training initiatives to support students, teachers and clinical mentors in their use of ePortfolios during clinical placements in higher healthcare education?

### Identifying Relevant Studies

Early in 2020, a preliminary search was conducted to explore the ePortfolio literature and to identify any reviews available on user training. Next, a list of search terms helped identify as many relevant articles as possible and combined searches were made of the terms: ‘ePortfolio’, ‘training’, ‘implementation’, ‘introduction’, ‘pedagogy’ and ‘learning model’. Wildcards were used to allow for different notations of the word ‘ePortfolio’. Four electronic databases were consulted: Web of Science, Elsevier Science Direct, ERIC and PubMed. The detailed search strategy is described in Online Resource 1.

### Selecting Studies

**Fig. 1:**
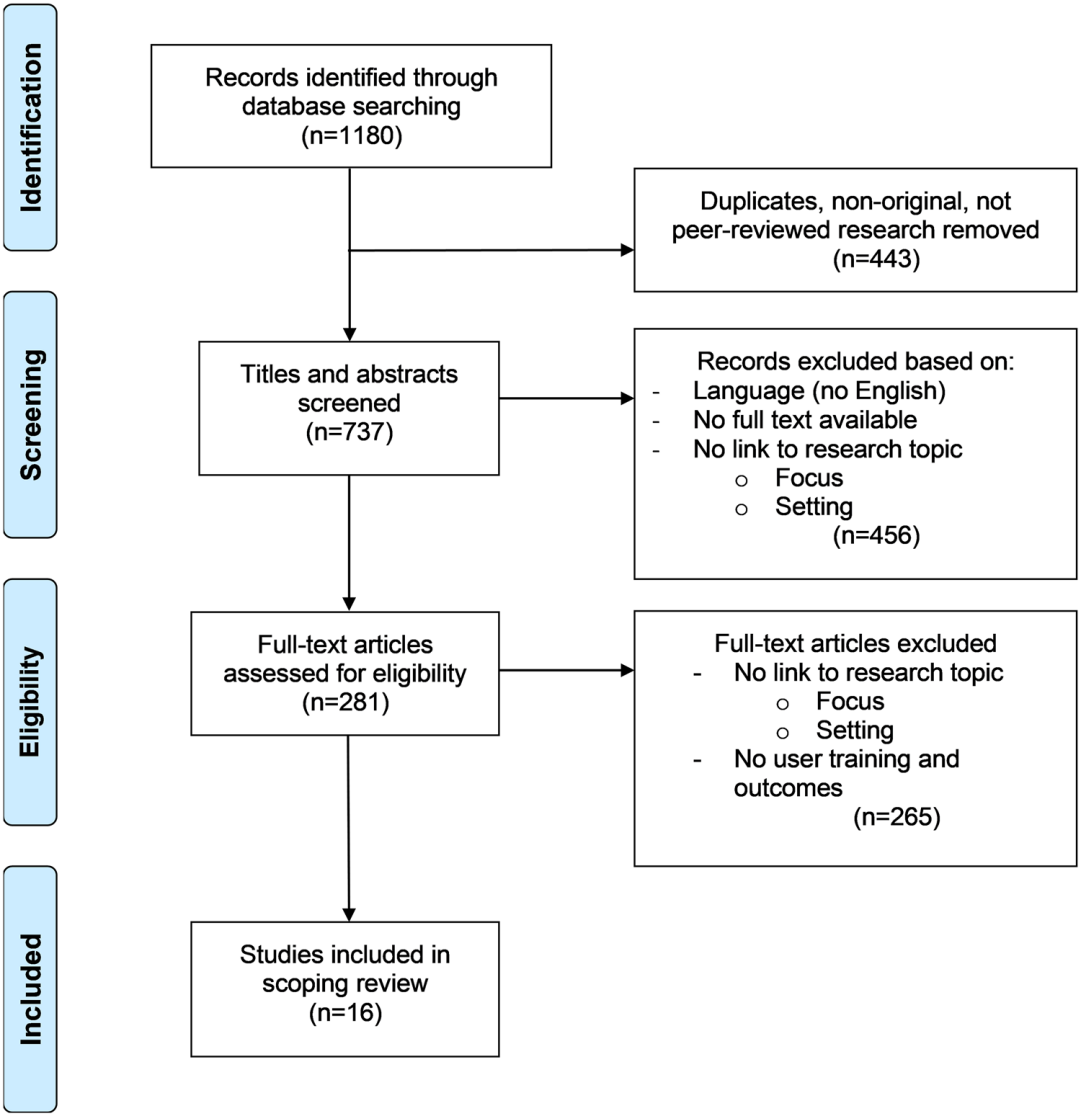
PRISMA flow diagram of the study selection stage

Three steps were followed to scrutinize the initial batch of articles. First, the articles were screened to remove duplicates and non-original research. Only peer-reviewed articles were selected to ensure the robustness of the studies included. Second, the titles and abstracts were screened for language (English and/or Dutch), availability of a full-text publication, and relevance to the research topic. Articles were excluded if (1) the study’s main focus was not related to ePortfolios (e.g. paper portfolios, games, electronic learning environments without a portfolio component), and (2) the study setting differed from clinical placements in higher healthcare education (e.g. a classroom environment, primary education, secondary education). Third, the full text of each article was read, again applying these two exclusion criteria, as some abstracts did not include any information about these criteria. The articles were also screened for information on ePortfolio user training and its evaluation. Only those articles adequately describing the user training and its outcomes (satisfaction, efficiency or effectiveness) were included. The selection stage followed the Preferred Reporting Items for Systematic Reviews and Meta-Analyses extension for Scoping Reviews (PRISMA-ScR) statement (see Online Resource 2) [31]. Figure 1 depicts this selection process.

### Charting the Data

In the fourth stage, key information items were extracted from the articles that were included. Using Microsoft Excel 16.0 (Excel 2016), a data charting form was developed based on the research question. Information items included authors’ names, year of publication, title, study location, educational program, program level, research aim, method, data collection and sections on user training and its outcomes. An overview of the items and related descriptive information for each article is provided in Online Resource 3. Two researchers pre-tested the data charting form on a set of randomly selected articles. The form, with minimal revisions, was used to extract information from all included articles. The first author charted the data for all articles that were included. The second author charted the data for a random sample of three articles to check for consistency and accuracy of extraction. Discrepancies were resolved through discussion. The extracted data of all articles were discussed with all the authors during intermediate consultation meetings. Points of discussion were clarified, and adjustments were made where necessary.

### Collating, Summarizing and Reporting the Results

Sections of the articles focusing on ePortfolio user training and its outcomes were categorized using thematic analysis [32]. After familiarizing with the data, the first author coded all extracted data and developed the training categories. These categories were generated inductively. In case of doubt, the other authors were consulted to reach a decision. This was done in about 50% of the codes. All authors approved the final list of codes and the actual coding of the article sections that focus on ePortfolio user training.

## Results

Database searches returned a total of 1180 articles which were screened and assessed for eligibility. This resulted in a set of 16 articles being included, all of which were published between 2008 and 2020. The educational programs in which the studies were conducted varied, with medicine (*n* = 6) and nursing (*n* = 3) being the most common. Sixty-nine percent (*n* = 11) of these educational programs were undergraduate programs. A summary of the characteristics of the studies included is presented in Table 1.

**Table 1:** Characteristics of the 16 included studies

**Characteristic**	**No. of studies (%)**
*Country*	
UK Australia New Zealand Canada Germany Indonesia Scotland South Africa USA United Arab Emirates	5 (32) 2 (13) 2 (13) 1 (6) 1 (6) 1 (6) 1 (6) 1 (6) 1 (6) 1 (6)
*Educational program*	
Medicine Nursing Dentistry Diagnostic radiography Midwifery Emergency health (paramedic) Orthodontics Physiotherapy + diagnostic radiography	6 (38) 3 (19) 2 (13) 1 (6) 1 (6) 1 (6) 1 (6) 1 (6)
*Program level*	
Undergraduate Postgraduate Graduate + undergraduate	10 (62) 5 (32) 1 (6)
*Research aim*	
To explore and examine perceptions of ePortfolio users To design, develop, implement and evaluate an ePortfolio To explore ePortfolio use	9 (56) 5 (32) 2 (13)
*Method*	
Mixed methods Qualitative Quantitative	10 (62) 4 (25) 2 (13)
*Data collection*	
Survey Survey + focus groups Survey + interviews Survey + focus groups + web analytics + document analysis Survey + focus groups + web analytics Interviews + focus groups + document analysis Interviews + document analysis Focus groups	5 (32) 4 (25) 2 (13) 1 (6) 1 (6) 1 (6) 1 (6) 1 (6)

### Characteristics of Included Studies

The research aims of studies included could be grouped into three categories: (1) to explore and examine perceptions of ePortfolio users; (2) to design, develop, implement and evaluate an ePortfolio; and (3) to explore ePortfolio use. Most studies (*n* = 9, 56%) fell in the first category. Sixty-two percent (*n* = 10) of the studies used a mixed method approach combining qualitative and quantitative research.

### User Training Initiatives and Their Outcomes

All studies (*n* = 16, 100%) reported on training initiatives for students. In six of the studies (38%), one or more of these initiatives were also aimed at clinical mentors (*n* = 4, 25%), teachers (*n* = 1, 6%) or both (*n* = 1, 6%). No training initiatives focused exclusively on teachers and/or clinical mentors.

Eight different user training subcategories were identified and were grouped into two main categories. The first main category contains general user training initiatives (*n* = 12, 75%), including face-to-face training (*n* = 10, 62%), the provision of online materials (*n* = 3, 19%), viewing other students’ artefacts, (*n* = 1, 6%), and the provision of a manual (*n* = 1, 6%). The second main category contains individual user training initiatives (*n* = 8, 50%), including feedback from teachers (*n* = 4, 25%), guidance from clinical mentors (*n* = 2, 13%), technical support (*n* = 2, 13%), and near-peer teaching supervision (*n* = 1, 6%). In six studies (38%), combinations of subcategories were observed to support ePortfolio users.

The information about training outcomes was either collected systematically by including one or two items referring to the user training in a survey or interview guide (*n* = 8, 50%), or emerged organically as a theme from collected data (*n* = 8, 50%). In the next section, we discuss the main user training categories and the related subcategories. We do this based on their occurrence in the literature, from most to least observed.

#### General User Training Initiatives

##### Face-to-Face Training

Face-to-face training was the most frequently used training approach but the outcomes were not favourable. Students, teachers and clinical mentors reported face-to-face training as being insufficient and only slightly useful [33–41]. Reasons for dissatisfaction related to the limited user-centered information [38], the short duration of the training [33–35, 38, 41, 42], and the inconvenient scheduling of training sessions, mostly too far ahead of the actual clinical placement [35, 42]. Different elements of improvement were suggested. The data pointed to a need for greater emphasis on how to set up an ePortfolio, how to use the ePortfolio system, what is expected at a concrete level and how to demonstrate good self-reflection skills [33, 34, 36, 38, 39, 41, 42]. Based on their experiences, users suggested organizing individual, well-planned, and continuous training [36, 38, 42].

##### Online Materials

Three studies described the provision of online materials (e.g. introduction section in the ePortfolio) as training initiative [38, 39, 47]. Students and clinical mentors did not find these online materials helpful. They did not get what was expected of them, and felt unsure on how to navigate the ePortfolio and what functions they could or were expected to use [38, 39]. In addition, they expressed concerns that the materials were not easily accessible to students who were not familiar with e-Learning and/or the ePortfolio concept [47].

##### Viewing Other Students’ Artefacts

Some ePortfolio designs supported peer learning by asking students to share their artefacts with other students. Only one study reported on the outcomes of this approach. In the study of Webb and Merkely [44], students (*n* = 40) were able to view their peers’ artefacts, and 57% of the students liked this functionality.

##### Manual

The study of Mason and Williams [47] was the only one describing the use of a manual that included information about where the ePortfolio could be accessed and assessment rubrics for each task. However, students complained of a lack of clear assessment guidelines, goals and requirements (e.g. word limits). This was considered a barrier to effectively completing their assessment task.

#### Individual User Training Initiatives

##### Feedback from Teachers

In general, students appreciated the feedback they received from teachers about submitted artefacts in their ePortfolio [43–45]. The following feedback-related outcomes could be identified: (1) promoting deep reflection and research [44, 45], (2) receiving formative feedback prior to summative assessment [46], and (3) enabling a personal, electronic dialogue with teachers about a student’s work [45]. However, students worried about the quality and quantity of the feedback. The following problems were discovered: (1) some teachers did not comment on uploaded artefacts which made students feel insecure about the status of their work, (2) some teachers were inconsistent in the timing of their feedback, and (3) some students indicated the feedback they received was too general and/or was lacking in detail [46].

##### Guidance from Clinical Mentors

In contrast to feedback from teachers, students expressed concerns about ePortfolio input from clinical mentors. Most students highlighted clinical mentors’ inability to offer useful guidance on the use of the ePortfolio [48]. However, in a small-scale study of Tonni and Oliver [38], students (*n* = 6) indicated that monthly meetings with their clinical mentor facilitated their reflective process.

##### Technical Support

In the study of Elshami et al. [43], students highlighted the importance of technical support while using an ePortfolio. However, in the study of Garrett et al. [35], the opinions of students and clinical mentors about the effectiveness of technical support were divided. Reasons for this disagreement were not reported or hard to discern.

##### Near-Peer Teaching Supervision

In the study of Vance et al. [39], postgraduate students supported undergraduate students with their ePortfolio use. The value of this near-peer teaching supervision was that postgraduate students were able to give practical insights on how to use the ePortfolio and what evidence of attainment to provide.

## Discussion

The aim of this scoping review was to consolidate evidence about ePortfolio user training initiatives and their outcomes in the context of clinical placements in higher healthcare education. To answer the research question, the fragmented literature about ePortfolio user training was mapped. The strength of the present study is that some key characteristics of ePortfolio user training could be identified to inform the design of training initiatives in the future.

The results show how ePortfolio research rarely focuses on user training. This fits the earlier observation of Tochel et al. [25]. Moreover, the available studies did not build on experimental research where different user training initiatives are offered to different groups of participants aimed at comparing different outcomes. Typically, data about training outcomes was collected using a limited number of items in a questionnaire or interview guide [35, 37, 38, 40, 43, 44, 46, 48], or the outcomes resulted as a theme from the data analysis without being specifically questioned [33, 34, 36, 39, 41, 42, 45, 47]. Therefore, in-depth information about the training outcomes and their impact on ePortfolio use is lacking. The minimal attention paid to user training in ePortfolio research is noticeable, given it is identified as a critical success factor for ePortfolio implementation [22]. More research is needed that focuses on ePortfolio user training initiatives and their outcomes as the core object of study; preferably based on experimental designs allowing to compare different training approaches and direct outcome variables.

In addition, the training outcomes were all reported based on user satisfaction instruments. This is consistent with results of other reviews that study training initiatives and their outcomes in healthcare education [49]. Since the aim of user training is to facilitate ePortfolio implementation and to respond to barriers related to the ability and (digital) skills of users using the tool [20], it is important to evaluate whether this aim is met. This study shows the need to conduct research that investigates the efficiency and effectiveness of user training, alongside user satisfaction.

Literature on the design of ePortfolio user training is rare. Nevertheless, this review detected different training designs and tried to generate new insights into how to improve future user training initiatives. Our analysis results indicate that general user training initiatives (e.g. face-to-face training, online materials, manuals) were considered less productive [33–42, 47] compared to individual initiatives (e.g. feedback from teachers, near-peer teaching supervision) [38, 39, 43–45, 47]. The main issues mentioned by training participants were that general initiatives did not provide the information they needed [38, 39, 47], were too short [33–35, 38, 41, 42], or came too far ahead of the actual use of the ePortfolio [35, 42]. These issues can be explained by not basing the design of these training initiatives in line with a fitting theoretical framework (e.g. action learning [50], self-directed learning [51], experiential learning [52], adult learning [53]). Those frameworks are built around instructional design features and expected outcomes, which could also be considered when designing and evaluating ePortfolio user training initiatives.

A final observation is that no training initiatives could be identified that focused exclusively on teachers and/or clinical mentors. The few training initiatives available for teachers and/or clinical mentors were also designed to train students [34, 35, 37–39, 41]. This suggests that designers of user training assume that the design features of training initiatives are equal for all user groups. This is unusual given that the purpose of ePortfolio use differs for each of these groups, implying different training needs. The provision of the same training initiatives to user groups with different training needs could explain the users’ dissatisfaction with these training initiatives. This highlights the need to set up more and better tailor-made training initiatives for teachers and clinical mentors that focus on their specific training needs.

### Limitations

Despite the systematic process followed in the literature review, some limitations must be pointed out. A first limitation is the selection of the literature search terms. We may have missed available studies on ePortfolio user training. This could be caused by the confusing terminology used when referring to this training. For example, user training—especially when aimed at teachers—is often labeled as ‘faculty development’ [16]. Though some of these studies could be traced, a future review could enrich the search set with additional concepts. A second limitation is related to the choice of inclusion and exclusion criteria. The inclusion of papers only written in English and/or Dutch leads to missing relevant papers published in other languages. The inclusion criterion of only including peer-reviewed research articles may also have affected available literature. Sometimes reports on user training are available but have been published as local reports, and are difficult to trace through standard scientific literature search tools. It is not the first time that a lack of empirical studies is reported in the context of training and professional development initiatives [54, 55]. A third limitation is the non-blind nature of the selection stage which carries the risk of bias. In order to avoid bias due to knowledge of authorship, institutions, journals and publication years, this identifying information should be removed prior to the selection stage [56, 57]. However, we strictly followed the PRISMA guidelines for scoping reviews, and to date, these guidelines do not include masking identifying information of yielded articles. Finally, the optional sixth step in a scoping review process, namely stakeholder consultation, was not implemented in the present study [30]. This is not uncommon even though stakeholder consultation may have enriched the results [58]. Such consultation is constrained because of the geographical setting, the language context, and the date of the initiatives set up in the past. Nevertheless, stakeholder consultation could be adopted as an appropriate starting point for user training design.

## Conclusions

This scoping review consolidated evidence from studies describing user training initiatives and their outcomes for the implementation of ePortfolios in support of competency development and assessment during clinical placements in higher healthcare education. The results provide an overview of the available evidence about ePortfolio user training and its outcomes, from which insights into the design, development and evaluation of such user training emerged. The researchers recommend grounding the design of training initiatives in line with a theoretical framework and propose an individual, ongoing training approach tailored to the training needs of specific user groups. Gaps in the literature on ePortfolio user training were also identified. Alongside the need for more research focusing on ePortfolio user training as the core object of study, there is a need for research into the efficiency and effectiveness of user training initiatives that complements evaluation of training initiatives in terms of user satisfaction. Future ePortfolio implementations can benefit from the insights provided by this review.

## Supplementary Information

Below is the link to the electronic supplementary material.

Supplementary file1 (PDF 77 KB)

Supplementary file2 (PDF 162 KB)

Supplementary file3 (PDF 109 KB)

## Data Availability

The datasets generated during and/or analysed during the current study are available from the corresponding author on reasonable request.
