# Supplementary material for: Training to Support ePortfolio Users During Clinical Placements: a Scoping Review
Source: Med Sci Educ. 2022 Jun 30;32(4):921–8. doi: 10.1007/s40670-022-01583-0 (PMC9411328; doi:10.1007/s40670-022-01583-0)
Supplement: Supplementary file 1 — Supplementary file1 (PDF 77 KB) [file 40670_2022_1583_MOESM1_ESM.pdf]

Online Resource 1. Search strategies for each database

Search strategy and yielded records per database

1. Web of Science (total = 566 records)

(e-portf* OR eportf*) (Topic) AND training (Topic)

194 records

(e-portf* OR eportf*) (Topic) AND implementation (Topic)

237 records

(e-portf* OR eportf*) (Topic) AND introduction (Topic)

61 records

(e-portf* OR eportf*) (Topic) AND pedagogy (Topic)

66 records

(e-portf* OR eportf*) (Topic) AND “learning model” (Topic)

8 records

2. Science Direct (total = 175 records)

Title, abstract, keywords:(e-portfolio OR e-portfolios OR eportfolio OR eportfolios) AND training

48 records

Title, abstract, keywords:(e-portfolio OR e-portfolios OR eportfolio OR eportfolios) AND implementation

81 records

Title, abstract, keywords:(e-portfolio OR e-portfolios OR eportfolio OR eportfolios) AND introduction

25 records

Title, abstract, keywords:(e-portfolio OR e-portfolios OR eportfolio OR eportfolios) AND pedagogy

20 records

Title, abstract, keywords:(e-portfolio OR e-portfolios OR eportfolio OR eportfolios) AND “learning model”

1 record

3. ERIC (total = 311 records)

noft((e-portf* OR eportf*)) AND noft(training)

78 records

Corresponding author: Sofie Van Ostaeyen; sofie.vanostaeyen@ugent.be

Department of Educational Studies, Faculty of Psychology and Educational Sciences, Ghent University, Henri Dunantlaan 2, 9000 Ghent, Belgium

noft((e-portf* OR eportf*)) AND noft(implementation)

138 records

noft((e-portf* OR eportf*)) AND noft(introduction)

28 records

noft((e-portf* OR eportf*)) AND noft(pedagogy)

62 records

noft((e-portf* OR eportf*)) AND noft(“learning model”)

5 records

4. PubMed (total = 124 records)

((e-portf*[Title/Abstract] OR eportf*)[Title/Abstract]) AND (training[Title/Abstract])

62 records

((e-portf*[Title/Abstract] OR eportf*)[Title/Abstract]) AND (implementation[Title/Abstract])

35 records

((e-portf*[Title/Abstract] OR eportf*)[Title/Abstract]) AND (introduction[Title/Abstract])

24 records

((e-portf*[Title/Abstract] OR eportf*)[Title/Abstract]) AND (pedagogy[Title/Abstract])

3 records

((e-portf*[Title/Abstract] OR eportf*)[Title/Abstract]) AND (“learning model”[Title/Abstract])

0 records

Corresponding author: Sofie Van Ostaeyen; sofie.vanostaeyen@ugent.be

Department of Educational Studies, Faculty of Psychology and Educational Sciences, Ghent University, Henri Dunantlaan 2, 9000 Ghent, Belgium
