# Supplementary material for: Training to Support ePortfolio Users During Clinical Placements: a Scoping Review
Source: Med Sci Educ. 2022 Jun 30;32(4):921–8. doi: 10.1007/s40670-022-01583-0 (PMC9411328; doi:10.1007/s40670-022-01583-0)
Supplement: Supplementary file 3 — Supplementary file3 (PDF 109 KB) [file 40670_2022_1583_MOESM3_ESM.pdf]

Online Resource 3. Descriptive information for included articles

First author (year)	Title	Location	Educational program	Program level	Research aim	Method	Data collection	User training initiatives	User group
Avila et al. (2016)	Evaluation of the free, open source software WordPress as electronic portfolio system in undergraduate medical education	Germany	Medicine	Under-graduate	To design, develop, implement and evaluate an ePortfolio	Mixed methods	Survey + focus groups	Face-to-face training	Students
Collins and O'Brien (2018)	Highly structured ePortfolio platform for bachelor of nursing students: Lessons learned in implementation	New Zealand	Nursing	Under-graduate	To design, develop, implement and evaluate an ePortfolio	Mixed methods	Survey + focus groups	Feedback from teachers	Students
De Swardt et al. (2019)	Implementing and evaluating an e-portfolio for postgraduate family medicine training in the Western Cape, South Africa	South Africa	Medicine	Post-graduate	To explore and examine perceptions of ePortfolio users	Mixed methods	Interviews + document analysis	Face-to-face training	Students + clinical mentors
Elshami et al. (2018)	Acceptability and potential impacts of innovative E-Portfolios implemented in E-Learning systems for clinical training	United Arab Emirates	Diagnostic radiography	Under-graduate	To explore and examine perceptions of ePortfolio users	Quantitative	Survey	Feedback from teachers + technical support	Students
Garrett et al. (2013)	Evaluation of an eportfolio for the assessment of clinical competence in a baccalaureate nursing program	Canada	Nursing	Under-graduate	To design, develop, implement and evaluate an ePortfolio	Mixed methods	Survey + focus groups + web analytics	Face-to-face training + technical support	Students + clinical mentors

Corresponding author: Sofie Van Ostaeyen; sofie.vanostaeyen@ugent.be

Department of Educational Studies, Faculty of Psychology and Educational Sciences, Ghent University, Henri Dunantlaan 2, 9000 Ghent, Belgium

Greviana et al. (2020)	Development of e-portfolio in undergraduate clinical dentistry: How trainees select and reflect on evidence	Indonesia	Dentistry	Under-graduate	To explore ePortfolio utilization	Qualitative	Interviews + focus groups + document analysis	Face-to-face training	Students + teachers
Haggerty and Thompson (2017)	The challenges of incorporating ePortfolio into an undergraduate nursing programme	New Zealand	Nursing	Under-graduate	To design, develop, implement and evaluate an ePortfolio	Qualitative	Survey	Face-to-face training	Students
Mason and Williams (2016)	Using ePortfolio's to assess undergraduate paramedic students: A proof of concept evaluation	Australia	Emergency health (paramedic)	Under-graduate	To explore and examine perceptions of ePortfolio users	Qualitative	Survey	Online materials + manual	Students
Peacock et al. (2011)	The transformative role of ePortfolios: Feedback in healthcare learning	Scotland	Physiotherapy + diagnostic radiography	Graduate + under-graduate	To explore ePortfolio utilization	Qualitative	Survey + focus groups	Feedback from teachers	Students
Pincombe et al. (2010)	ePortfolio in midwifery practice: "The way of the future"	Australia	Midwifery	Under-graduate	To design, develop, implement and evaluate an ePortfolio	Mixed methods	Survey + interviews	Face-to-face training	Students
Tailor et al. (2014)	Opinions of the ePortfolio and workplace-based assessments: A survey of core medical trainees and their supervisors	United Kingdom	Medicine	Post-graduate	To explore and examine perceptions of ePortfolio users	Quantitative	Survey	Face-to-face training	Students + clinical mentors
Tonni and Oliver (2013)	Acceptability of a reflective e-portfolio instituted in an orthodontic specialist programme: A pilot study	United Kingdom	Orthodontics	Post-graduate	To explore and examine perceptions of ePortfolio users	Mixed methods	Survey	Face-to-face training + Online materials + Guidance	Students + teachers + clinical mentors

Corresponding author: Sofie Van Ostaeyen; sofie.vanostaeyen@ugent.be

Department of Educational Studies, Faculty of Psychology and Educational Sciences, Ghent University, Henri Dunantlaan 2, 9000 Ghent, Belgium

								from clinical mentors	
Vance et al. (2013)	Evaluation of an established learning portfolio	United Kingdom	Medicine	Post-graduate	To explore and examine perceptions of ePortfolio users	Mixed methods	Survey	Guidance from clinical mentors	Students
Vance et al. (2017)	Longitudinal evaluation of a pilot e-portfolio-based supervision programme for final year medical students: Views of students, supervisors and new graduates	United Kingdom	Medicine	Under-graduate	To explore and examine perceptions of ePortfolio users	Mixed methods	Survey + interviews	Face-to-face training + online materials + near peer teaching supervision	Students + clinical mentors
Vernazza et al. (2011)	Introduction of an e-portfolio in clinical dentistry: Staff and student views	United Kingdom	Dentistry	Under-graduate	To explore and examine perceptions of ePortfolio users	Mixed methods	Survey + focus groups	Face-to-face training	Students
Webb and Merkley (2012)	An evaluation of the success of a surgical resident learning portfolio	USA	Medicine	Post-graduate	To explore and examine perceptions of ePortfolio users	Mixed methods	Survey + focus groups + web analytics + document analysis	Feedback from teachers + viewing other students' artefacts	Students

Corresponding author: Sofie Van Ostaeyen; sofie.vanostaeyen@ugent.be

Department of Educational Studies, Faculty of Psychology and Educational Sciences, Ghent University, Henri Dunantlaan 2, 9000 Ghent, Belgium
